# Expression of vascular endothelial growth factor mRNA in non-small-cell lung carcinomas

**DOI:** 10.1038/sj.bjc.6690058

**Published:** 1999-01

**Authors:** G Fontanini, L Boldrini, S Chinè, F Pisaturo, F Basolo, A Calcinai, M Lucchi, A Mussi, C A Angeletti, G Bevilacqua

**Affiliations:** Department of Oncology, Division of Pathology, University of Pisa, Italy; Department of Surgery, Service of Thoracic Surgery, University of Pisa, Italy

**Keywords:** vascular endothelial growth factor, non-small-cell lung cancer, reverse transcription polymerase chain reaction, prognosis

## Abstract

The vascular endothelial growth factor (VEGF) has been shown to be strictly related to vascular permeability and endothelial cell growth under physiological and pathological conditions. In tumour development and progression, VEGF plays a pivotal role in the development of the tumoral vascular network, and useful information in the progression of human cancer can be obtained by analysing the vascular endothelial growth factor expression of the tumours. In this study, we investigated the vascular endothelial growth factor transcript expression in non-small-cell lung carcinomas to evaluate the significance of this factor in a group of cancers in which the vascular pattern has been shown to significantly affect progression. Surgical samples of 42 patients with NSCLC were studied using reverse transcription polymerase chain reaction (PCR) analysis and in situ hybridization. Thirty-three out of 42 cases (78.6%) showed VEGF transcript expression predominantly as transcripts for the secretory forms of VEGF (isoforms 121 and 165). In situ hybridization, performed on 24 out of 42 samples, showed that the VEGF transcript expression was in several cases present in the cytoplasm both of neoplastic and normal cells, even if the VEGF mRNA was less expressed in the corresponding non-tumoral part. The VEGF 121 expression was associated with hilar and/or mediastinal nodal involvement (*P* = 0.02), and, taken together, the VEGF isoforms were shown to significantly influence overall (*P* = 0.02) and disease-free survival (*P* = 0.03). As a regulator of tumour angiogenesis, VEGF may represent a useful indicator of progression and poor prognosis in non-small-cell lung carcinomas. © 1999 Cancer Research Campaign


*British Journal of Cancer* (1999) **79**(2), 363–369
© 1999 Cancer Research Campaign

### Expression of vascular endothelial growth factor mRNA in non-small-cell lung carcinomas

**G Fontanini[1], L Boldrini[1], S Chinè[1], F Pisaturo[1], F Basolo[1], A Calcinai[1], M Lucchi[2], A Mussi[2], CA Angeletti[2] and G Bevilacqua[1]**

[1]Department of Oncology, Division of Pathology and [2]Department of Surgery, Service of Thoracic Surgery, University of Pisa, Italy

**Summary** The vascular endothelial growth factor (VEGF) has been shown to be strictly related to vascular permeability and endothelial cell growth under physiological and pathological conditions. In tumour development and progression, VEGF plays a pivotal role in the development of the tumoral vascular network, and useful information in the progression of human cancer can be obtained by analysing the vascular endothelial growth factor expression of the tumours. In this study, we investigated the vascular endothelial growth factor transcript expression in non-small-cell lung carcinomas to evaluate the significance of this factor in a group of cancers in which the vascular pattern has been shown to significantly affect progression. Surgical samples of 42 patients with NSCLC were studied using reverse transcription polymerase chain reaction (PCR) analysis and in situ hybridization. Thirty-three out of 42 cases (78.6%) showed VEGF transcript expression predominantly as transcripts for the secretory forms of VEGF (isoforms 121 and 165). In situ hybridization, performed on 24 out of 42 samples, showed that the VEGF transcript expression was in several cases present in the cytoplasm both of neoplastic and normal cells, even if the VEGF mRNA was less expressed in the corresponding non-tumoral part. The VEGF 121 expression was associated with hilar and/or mediastinal nodal involvement ($P = 0.02$), and, taken together, the VEGF isoforms were shown to significantly influence overall ($P = 0.02$) and disease-free survival ($P = 0.03$). As a regulator of tumour angiogenesis, VEGF may represent a useful indicator of progression and poor prognosis in non-small-cell lung carcinomas.

**Keywords:** vascular endothelial growth factor; non-small-cell lung cancer; reverse transcription polymerase chain reaction; prognosis

The vascular endothelial growth factor (VEGF), a homodimeric glycoprotein of relative molecular mass 45 000, has been recently identified as a vascular permeability factor, and as an endothelial cell-specific mitogenic factor in vivo. By alternating splicing of mRNA, four different isoforms with 121, 165, 189 and 206 amino acids may be identified (Tischer et al, 1991; Houck et al, 1991). These isoforms have different bioavailability, owing to their different heparin-binding activity: VEGF-121 fails to bind heparin and is secreted as a freely soluble protein. VEGF-165 is a basic, heparin-binding protein and it is also secreted, but to a lesser degree than the VEGF-121 isoform. The longer isoforms have a greater affinity to heparin and are stably incorporated in the extracellular matrix.

Several studies have shown that VEGF mRNA is expressed in a variety of human tumours including renal (Brown et al, 1993), mammary (Brown et al, 1995; Toi et al, 1996; Yoshiji et al, 1996), colonic (Takahashi et al, 1995), œsophageal (Inoue et al, 1997), gastric (Maeda et al, 1996), hepatocellular (Suzuki et al, 1996), ovarian (Abu-Jawdeh et al, 1996) and lung (Mattern et al, 1995, 1996; Otha et al, 1996; Volm et al, 1996*a*, 1996*b*) carcinomas. Because the VEGF expression has been shown to be strictly related to neovascularization and poor prognosis in different types of human cancers (Mattern et al, 1995; Takahashi et al, 1995; Volm et al, 1996*a*), it may be useful to analyse the VEGF mRNA expression with particular regard to its different isoforms. The role of VEGF in the behaviour of human cancer could be particularly interesting because it might represent an excellent target for the development of new anti-tumour strategies, based on the inhibition of tumour angiogenesis (Martiny-Baron et al, 1995). In this respect, non-small-cell lung carcinoma, in which neoangiogenesis seems to be an important adjunctive prognostic indicator (Macchiarini et al, 1992; Fontanini et al, 1995; Angeletti et al, 1996; Harpole et al, 1996) providing useful information on new therapeutic approaches, may represent an interesting human model to investigate the influence of VEGF in its development and progression.

In this study, we analysed the VEGF mRNA expression by reverse transcription polymerase chain reaction (RT-PCR) and in situ hybridization to better define the role of this angiogenic factor in the behaviour of non-small-cell lung cancer (NSCLC).

### MATERIALS AND METHODS

### Tissue samples

Non-small-cell lung cancer tissues obtained from 42 patients who received surgery at the S. Chiara Hospital of Pisa University, Italy, between 1991 and 1994 were frozen and stored at –80°C. Eighteen fragments from surrounding normal tissues were also analysed. There were 38 men and four women (mean age 62.4, range 42–77). Median follow-up was 39.5 months (range 22–59). The patients presented no detectable metastases in distant organs at the time of surgery. Tumour samples were formalin-fixed and

*Received 26 January 1998*
*Revised 1 May 1998*
*Accepted 6 May 1998*

*Correspondence to:* G Fontanini, Department of Oncology, Division of Pathology, via Roma, 57, 56126 Pisa, Italy

**Table 1** Univariate analysis of the association between clinicopathological parameters and overall survival in 42 cases of NSCLC

| Patient and tumour characteristics | No of cases | Two-sided *P*-value* |
|---|---|---|
| Sex | | |
| Men | 37 | 0.4 |
| Women | 5 | |
| Age | | |
| ≤ 64 | 24 | 0.6 |
| > 64 | 18 | |
| Histology | | |
| Squamous | 21 | |
| Adenocarcinoma | 15 | 0.2 |
| Anaplastic large cell | 3 | |
| Bronchioloalveolar | 3 | |
| Tumour size | | |
| T1 | 10 | |
| T2 | 27 | 0.04 |
| T3 | 5 | |
| Node status | | |
| N0 | 25 | |
| N1 | 6 | 0.02 |
| N2 | 11 | |
| Stage | | |
| S1 | 24 | |
| S2 | 5 | 0.03 |
| S3 | 13 | |

*Cox's F-model.

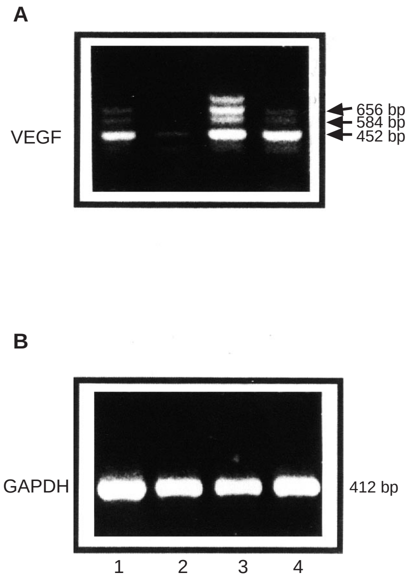

**Figure 1** (**A**) Identification by RT-PCR of VEGF isoforms expressed in human lung carcinomas. Expected VEGF amplification products of 452, 584 and 656 bp correspond to VEGF isoforms VEGF-121, VEGF-165 and VEGF-189 respectively. (**B**) A single 412-bp band amplified with primers specific for GAPDH with the same cDNAs under identical conditions. Lanes 1, 2, 3, 4: four NSCLC samples paraffin-embedded for histological examination and immunohistochemical analysis of the microvascular endothelium. Tumour fragments were analysed according to the WHO histological classification (World Health Organization, 1982) and the guidelines of the American Joint Committee for Cancer Staging (American Joint Committee on Cancer, 1992) with regard to pathological studies.

### Microvessel detection and counting

In all cases, the number of microvessels in the tumours was determined after highlighting vessel endothelium with a monoclonal antibody (QB-END Novocastra Laboratories, Newcastle, UK) directed against the CD34 antigen. A single microvessel was defined as any brown, immunostained endothelial cell separated from adjacent microvessels, tumour cells and connective tissue elements. In each sample, the three most intense regions of neovascularization under low microscopic power (× 10 objective lens and × 10 ocular lens) were identified. A × 250 field (× 25 objective lens and × 10 ocular lens; 0.74 $mm^2$ per field) in each of these three areas was then counted, and the average count of the three fields was recorded. Large vessels with thick, muscular walls were excluded from the counts. The presence of a lumen was not required to identify a microvessel.

### RT-PCR analysis

Total RNA was extracted from frozen lung tissues using a RNA extraction reagent, Ultraspec RNA, according to the standard acid–guanidium–phenol–chloroform method. Five micrograms of total RNA were reverse transcribed at 42°C for 60 min in a total 20-μl reaction volume using a 1st-Strand cDNA synthesis kit (Clontech Laboratories, Palo Alto, CA, USA). cDNA was incubated at 95°C for 5 min to inactivate the reverse transcriptase, and served as template DNA for 30 rounds of amplification using the Gene Amp PCR System 2400 (Perkin-Elmer Applied Biosystems, CA, USA). PCR was performed in a standard 50-μl reaction mixture consisting of 10 mM Tris-HCl, 50 mM potassium chloride, 1.5 mM magnesium chloride (pH 8.3), 0.2 mM dNTPs, 50 pmol of each sense and antisense primer and 2.5 U of Amplitaq DNA polymerase (Perkin-Elmer Applied Biosystems). Amplification was performed for 30 sec at 94°C, 1 min at 55°C and 1 min at 72°C. Finally, an additional extension step was carried out for 2 min. As negative control, the DNA template was omitted in the reaction. The amplification products were separated on 1.5% agarose gels and visualized by ethidium bromide staining. PCR primers for VEGF cDNA were as follows: forward primer, 5′-TGGATCCATGAACTTTCTGCTGTC-3′; reverse primer, 5′-TCACCGCCTTGGCTTGTCACAT-3′ according to the VEGF gene structure. Three kinds of PCR product of 656 bp, 584 bp and 452 bp encoding VEGF isoforms VEGF –189, VEGF –165 and VEGF –121, respectively, were obtained. For GAPDH, the forward primer was 5′-CGATGCTGGCGCTGAGTAC-3′ and the reverse primer was 5′-CGTTCAGCTCAGGGATGACC-3′ (Wizigmann-Voos et al, 1995). The presence of a single 412-bp band amplified with primers specific for GAPDH with the same cDNAs was used as internal control under identical conditions.

### In situ hybridization

In situ hybridization was performed in 24 out of 42 cases using a 40-bp 3′-biotin-labelled, single-stranded synthetic probe (Oncogene Science, Manhasset, NY, USA) to identify the VEGF mRNA sequence. Briefly, formalin-fixed and paraffin-embedded tumour samples were cut, mounted on APES-coated slides and

 © Cancer Research Campaign 1999

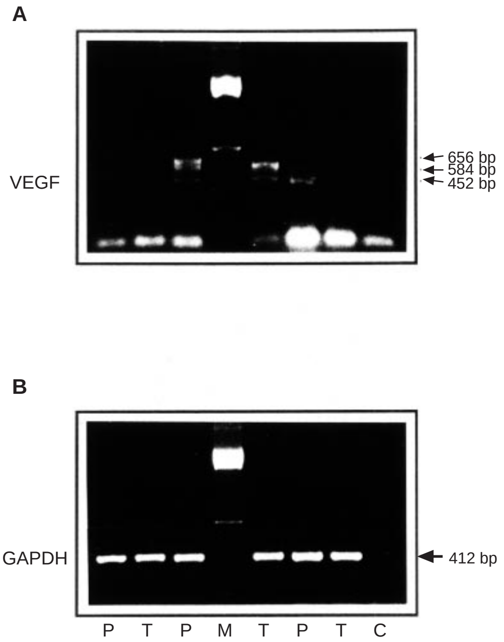

**Figure 2** (**A**) The results of the electrophoretic analysis of PCR products for VEGF in three representative parenchyma/tumour (P/T) couples: in the first couple, both normal lung tissue and tumoral part were negative for the VEGF expression; in the second couple, the VEGF signal was revealed in both tissues although with different patterns of VEGF isoforms; in the third couple, only the surrounding normal lung tissue showed VEGF expression. Lane M: 100-b-p ladder (Pharmacia Biotech). Lane C: negative control, with no cDNA added to PCR mixture. (**B**) a single 412-bp band amplified with primers specific for GAPDH with the same cDNAs under identical conditions baked at 37°C overnight. Slides were dewaxed with xylene and rehydrated through graded alcohols at room temperature. After washing in 0.1% diethylpyrocarbonate (DEPC)-treated water, the slides were soaked in 2 × standard saline citrate (SSC) (0.3 M sodium chloride, 30 mM sodium citrate) at 60°C for 10 min followed by digestion with proteinase K (20 mg ml$^{-1}$ in 0.05 M Tris, pH 7.6) at 37°C for 1 h. Sections were then fixed in 0.4% paraformaldehyde/phosphate-buffered saline (PBS) at 4°C for 20 min. After brief washing in DEPC-treated water and incubation in prehybridization buffer (37°C for 1 h), a hybridization solution containing labelled probe (0.2 mg ml$^{-1}$) was applied to the sections, coverslips were added and the slides were incubated in a moist chamber overnight at 37°C. After hybridization with antisense probe, the coverslips were removed and the slides were washed sequentially in 4 × SSC (0.6 M sodium chloride, 60 mM sodium citrate) containing 30% formamide, 2 × SSC at 37°C, and 0.2 × SSC (30 mM sodium chloride, 3 mM sodium citrate) at room temperature. After immersion in 1 × modified Tris-buffered saline (TBS) Tween 20 [50 mM Tris (pH 7.6), 150 mM sodium chloride and 0.5% Tween 20], slides were incubated with streptavidin–HRP (horseradish peroxidase) for 30 min at 37°C in a humid chamber; a signal amplification technique was performed by incubation with biotinylated-tyramide for 10 min at room temperature. The colouring reaction was performed by aminoethylcarbazole (AEC). The slides were rinsed in water, counterstained with haematoxylin, and mounted with aqueous mounting. Two controls were used to check the specificity of the hybridization signal: a RNAAse pretreatment of the tissue sections and a substitution of the antisense probe with a biotin-labelled random probe. This random probe was a 40-bp single-stranded synthetic oligonucleotide with randomized sequence, except for the 3′ end deoxyadenosine base, and was used under the same stringency conditions.

### Statistical analysis

All statistical analyses were carried out by the Statistica software system. Survival curves were obtained by the Kaplan–Meier method, and survival rate was assessed by Cox's *F*-model. The relationship between VEGF expression and clinicopathological characteristics was analysed by the contingency tables.

### RESULTS

### Patients and tumour characteristics

The most common histological type was squamous cell carcinoma (50%) followed by adenocarcinoma (35.7%), large-cell anaplastic carcinoma (7.15%) and bronchioloalveolar carcinoma (7.15%). With respect to tumour size, ten (23.8%) tumours were classified as T1, 27 (64.3%) were classified as T2 and five (11.9%) were classified as T3. Six (14.3%) cancers showed metastatic involvement of the hilar lymph nodes (N1), whereas mediastinal lymph nodes (N2) were involved in 11 (26.1%) cancers. No metastatic involvement (N0) was present in 25 (59.5%) patients. Most cases were classified as stage I (24; 57.1%), five (11.9%) were classified as stage II and 13 (31%) were classified as stage III. Nineteen (45.2) patients relapsed during follow-up. At the time of analysis, 28 (66.6%) patients were alive, whereas 14 (33.4%) patients were dead.

### Clinicopathological characteristics and survival

Among the clinicopathological parameters, greater tumour size (test for trend, two-sided $P$ = 0.04), metastatic nodal involvement (test for trend, two-sided $P$ = 0.02) and advanced stage (test for trend, two-sided $P$ = 0.03) were significantly associated with a worse overall survival (Table 1).

### RT-PCR analysis of VEGF mRNA in normal and NSCLC tissues

RT-PCR analysis revealed that VEGF mRNA was expressed in the lung tumours of 33 out of 42 patients examined (78.6%). Three kinds of amplified cDNAs (VEGF-121, VEGF-165, VEGF-189) were detected. Amplification products corresponding to VEGF 206 were undetectable. Thirteen out of 42 (31%) cases expressed the VEGF-189 isoform; 24 out of 42 (57.1%) expressed the VEGF-165 isoform and 32 out of 42 (76.1%) showed the VEGF-121 splice. Nine out of 33 (27.3%) expressed the VEGF-121 isoform alone; in 23 out of 33 (69.7%) cases, the two secretory forms (VEGF-121 and VEGF-165) were concomitantly detected. Of the samples, 39.4% showed all three isoforms, whereas only one case (3.03%) was positive for VEGF-165 alone. Figure 1 shows the results of the electrophoretic analysis of PCR products in representative cases. In 18 cases, fragments of surrounding normal lung tissue were also examined. Seventeen out of 18 (94.4%) cases showed VEGF mRNA positivity with preponderant expression of the 121 and 165 isoforms. One negative case showed no VEGF mRNA expression, even in the tumoral part. Figure 2 shows the results of the electophoretic analysis of PCR products for VEGF in three representative parenchyma/tumour (P/T) couples.

### In situ hybridization

VEGF antisense probes were used to perform in situ hybridization in 24 of the 42 cases analysed. As shown in Figure 3, the VEGF

© Cancer Research Campaign 1999

mRNA expression was widely detected in the cytoplasm of tumour cells in 75% of the cases (Figure 3A). In 21 of the 24 cases analysed, surrounding lung tissue was concomitantly present and the VEGF mRNA expression was revealed in the cytoplasm of hyperplastic alveolar cells (Figure 3B) and/or normal columnar cells lining submucosal glands (Figure 3C) in 47.6% of cases; moreover, several inflammatory cells expressed VEGF transcript (Figure 3D). No VEGF mRNA expression was detected in the endothelial cells. Figure 4A and B shows in situ hybridization with both antisense and random probes in one case of lung adenocarcinoma.

### VEGF mRNA expression and clinicopathological parameters

The VEGF mRNA expression was compared with the clinical and histological features of the tumours. The comparison between the different isoforms of VEGF and clinicopathological characteristics of the tumours is shown in Table 2. No association was found between VEGF transcript and age or sex of the patients, nor with histology and size of the tumour. A significant association was present between the VEGF-121 isoform and nodal status. In fact, of the 17 tumours with metastatic nodal involvement at the moment of resection, 16 were positive for the VEGF-121 form ($P = 0.02$, chi-squared test).

### VEGF mRNA expression and survival

The relation between the VEGF mRNA expression and survival was also investigated. Patients with VEGF-mRNA-negative tumours showed better disease-free and overall survival ($P = 0.03$, $P = 0.02$) than those patients with VEGF-mRNA-positive tumours (Figure 5A and B). The prognostic impact of the different isoforms was also analysed. The VEGF-mRNA-165 expression significantly affected overall survival ($P = 0.01$) (Figure 6), in contrast to the other two isoforms.

### VEGF mRNA expression and microvessel density

To examine a possible relationship between VEGF mRNA expression and microvessel density, the data were analysed by the *T*-test for independent samples. Mean vessel count (± s.d.) was higher in VEGF-mRNA-positive tumours (29.8 ± 23.5) than in VEGF-mRNA-negative tumours (23 ± 11.4), although this difference was not statistically significant ($P = 0.49$). Similar results were obtained

A B C D

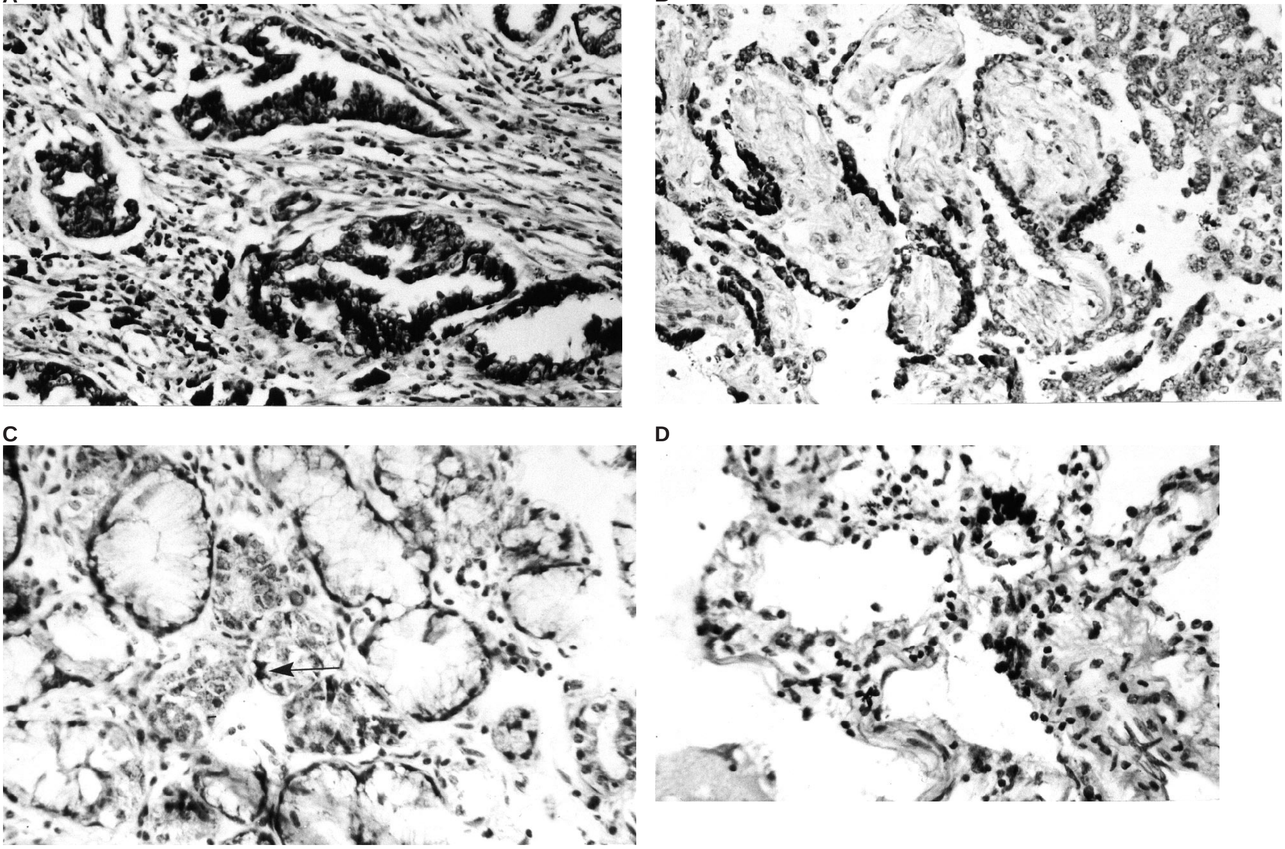

**Figure 3** Non-radioactive in situ hybridization in a sample of non-small-cell lung cancer (**A**). Normal alveolar cells (**B**), epithelium lining sub-mucosal glands (**C**) (arrows) and inflammatory cells (**D**) in a sample of normal lung tissue expressing VEGF mRNA. The sections were hybridized with antisense VEGF probe

© Cancer Research Campaign 1999

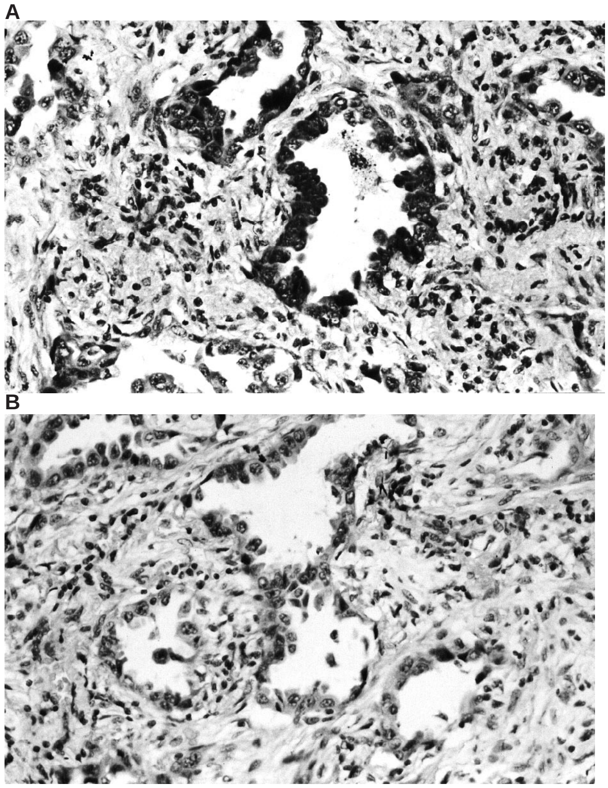

**Figure 4** Non-radioactive in situ hybridization with both antisense (**A**, arrows) and random probes (**B**) in one case of lung adenocarcinoma when the association between vascular density and the three different isoforms of VEGF mRNA was analysed (data not shown).

### DISCUSSION

VEGF has been identified recently as a secreted endothelial cell-specific mitogen able to stimulate angiogenesis in vivo (Ferrara, 1995; Shibuya et al, 1995). Four different isoforms (VEGF-121, VEGF-165, VEGF-189, VEGF-206) have been described in tumours as alternative splicing of a single gene (Tiesher et al, 1991). Transcripts encoding the three shorter forms, 121, 165 and 189, have been detected in the majority of tumour cells expressing the VEGF gene. VEGF-121 and VEGF-165 isoforms are efficiently secreted and mostly stimulate the mitogenic properties of endothelial cells. On the contrary, the longer isoforms (VEGF-189 and VEGF-206) are generally cell-associated and linked to vascular permeability (Ferrara, 1995). However, the significance of the various isoforms has not been completely explained. According to the different availability and affinity with their receptors, they could be differently involved in the development of tumoral angiogenesis.

### VEGF expression in NSCLC

Several studies have analysed the VEGF expression in human cancers such as mammary (Brown et al, 1995; Toi et al, 1996; Yoshiji et al, 1996), urinary (Brown et al, 1993), gastric (Maeda et al, 1996), colonic (Takahashi et al, 1995), ovarian (Abu-Jawdeh et al, 1996) hepatocellular (Suzuki et al, 1996) and lung (Mattern et al, 1995, 1996; Otha et al, 1996; Volm et al, 1996*a*, 1996*b*) carcinomas. In these studies, mRNA analysis showed a predominant expression of the VEGF secretory forms 121 and 165. According

**Table 2** VEGF mRNA expression according to clinicopathological characteristics

| Variables | VEGF-189 | | *P*-value | VEGF-165 | | *P*-value | VEGF-121 | | *P*-value |
|---|---|---|---|---|---|---|---|---|---|
| | Positive | Negative | | Positive | Negative | | Positive | Negative | |
| Age (mean ± s.d.) | 61.4 ± 7 | 62.8 ± 8 | NS | 62.1 ± 9 | 62.8 ± 7 | NS | 61.7 ± 7 | 64.5 ± 8 | NS |
| Sex | | | | | | | | | |
| Men | 11 | 26 | | 21 | 16 | | 28 | 9 | |
| | | | NS | | | NS | | | NS |
| Women | 2 | 3 | | 3 | 2 | | 4 | 1 | |
| Histology | | | | | | | | | |
| Squamous | 9 | 12 | | 15 | 6 | | 18 | 3 | |
| Adeno | 4 | 11 | | 8 | 7 | | 10 | 5 | |
| | | | NS | | | NS | | | NS |
| Anaplastic | 0 | 3 | | 1 | 2 | | 3 | 0 | |
| Br/Al | 0 | 3 | | 0 | 3 | | 1 | 2 | |
| T-status | | | | | | | | | |
| T1 | 4 | 6 | | 4 | 6 | | 8 | 2 | |
| T2 | 7 | 20 | NS | 16 | 11 | NS | 19 | 8 | NS |
| T3 | 2 | 3 | | 4 | 1 | | 5 | 0 | |
| N-Status | | | | | | | | | |
| N0 | 7 | 18 | | 12 | 13 | | 16 | 9 | |
| | | | NS | | | NS | | | 0.02 |
| N1–2 | 6 | 11 | | 12 | 5 | | 16 | 1 | |

*© Cancer Research Campaign 1999*

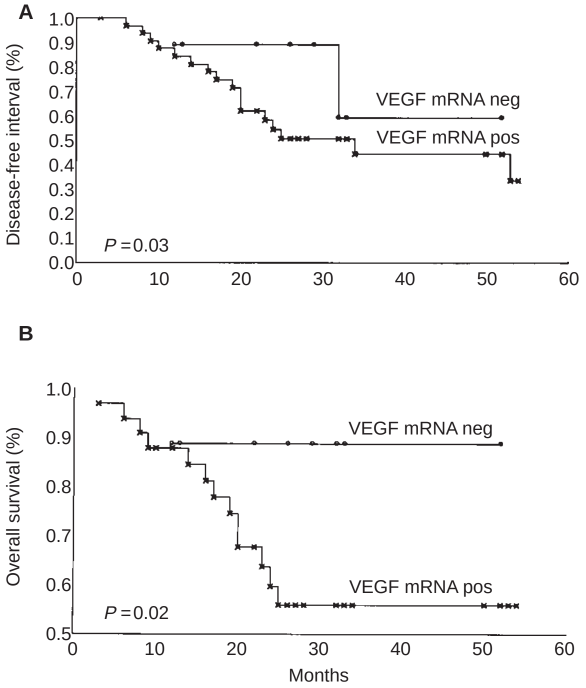

**Figure 5** Disease-free (**A**) and overall survival (**B**) curves for 42 patients with non-small-cell lung cancer. The Kaplan–Meier method was used to estimate disease-free and overall survival, and the Cox's *F*-test was used to compare the curves. Disease-free and overall survival were affected by VEGF mRNA expression which was stratified in two groups: nine cases negative and 33 cases positive

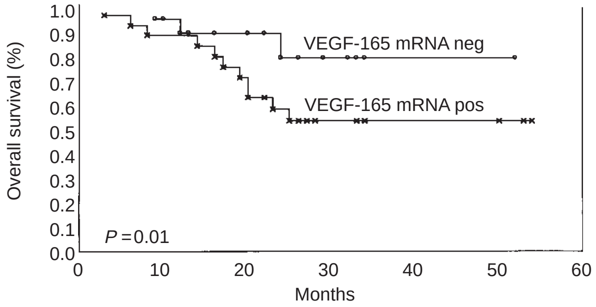

**Figure 6** Overall survival curve in patients with non-small-cell lung cancer according to VEGF-mRNA-165 isoform. The Kaplan–Meier method was used to estimate overall survival, and the Cox's *F*-test was used to compare the curves. Overall survival was affected by VEGF-mRNA-165 isoform expression which was stratified in two groups: 18 cases negative and 24 cases positive to these data, the results of our RT-PCR analysis revealed a similar pattern of the VEGF expression. Moreover, in our study, the VEGF expression was detected in almost all cases of the adjacent non-neoplastic tissues. This agrees with the putative biological role of VEGF in that it maintains the permeability of normal lung tissues. The frequent expression of VEGF mRNA in non-neoplastic samples found in our series may also be explained by the presence of inflammatory changes, which are often detected concomitantly with this type of neoplastic lesion. This is confirmed by in situ hybridization results which showed a clear expression of VEGF mRNA in the cytoplasm of tumour cells, and even in normal and inflammatory elements as well as in the hyperplastic type II alveolar cells which are usually present in the inflammatory response. As regards normal tissue, VEGF mRNA positivity was predominantly found in the cytoplasm of alveolar cells. Only a few cases of epithelial cells lining submucosal glandulae showed VEGF expression. Thus, the VEGF expression detected in squamous and adenocarcinomas (arising from bronchial-lining epithelium) may actually be considered as strictly related to the progression of NSCLC with likely prognostic implications; conversely, because two of the three cases of bronchioloalveolar carcinoma were VEGF negative, we can suppose that in the alveolar-arising tumours, such as the bronchioloalveolar tumours, VEGF will have a different role. However, further analyses by quantitative PCR will be necessary to confirm the higher mRNA VEGF levels in tumour cells compared with normal tissues. The different percentages of the VEGF cases expressing VEGF in tumour samples by using RT-PCR and in situ hybridization (ISH) (94.4% by PCR vs 75% by ISH) may probably be due to the fact that with the RT-PCR method also very small amounts of VEGF mRNA are detected, whereas with ISH greater amounts of transcript are necessary for the signal to be observed.

### VEGF expression and tumour progression

Several studies have analysed the relationship between VEGF expression, clinicopathological features and survival (Takahashi et al, 1995, 1996; Maeda et al, 1996; Toi et al, 1996; Volm et al, 1996*a*; Inoue et al, 1997) mostly by using immunohistochemical techniques. In lung cancer, the VEGF mRNA expression has been studied by Otha et al (1996) using RT-PCR assay. Interesting data concerning the prognostic impact of VEGF have been reported. However, their analysis included both non-small- and small-cell lung carcinomas and only the contribution of the VEGF-121 isoform was investigated. In our study, we confirmed the data by Otha et al (1996) about the prognostic impact of VEGF on overall survival. However, our study provides further information about the influence of VEGF on the behaviour of this type of cancer. In fact, a significant association was noticed between the VEGF-165 isoform and survival, with no significant association between the 121 and 189 isoforms and survival when the latter were individually analysed. This suggests the possibility of a predominant role of isoform 165 in the progression of this type of cancer. However, the significant association we found between the VEGF-121 isoform and nodal metastatic involvement underlines the importance of VEGF soluble isoforms as markers of aggressiveness in this tumour. Our recent analysis concerning the relation between high-VEGF protein expression and poor prognosis in a series of 107 NSCLC (Fontanini et al, 1997) agrees with the data obtained with our RT-PCR analysis. A polyclonal antibody directed against the 121, 165 and 189 amino acid splice variants was used to recognize the VEGF antigen. This suggests that the cytoplasmic antigen detected in cancer cells could be that of the secretory isoforms as confirmed by RT-PCR analysis, which shows a predominant expression of 121 and 165 isoforms. Moreover, in the present study, the soluble isoforms are those related to poor prognosis and metastasis, confirming the data of our previous study using the immunohistochemical approach. However, these preliminary data need further investigations in a larger and prospective series of patients to better understand the real role of the soluble isoforms in the outcome of NSCLC, and before considering the expression of the VEGF as a prognostic parameter in this type of cancer.

© Cancer Research Campaign 1999

### VEGF expression and vascular count in NSCLC

To investigate the influence of VEGF as a regulatory factor of tumour angiogenesis, we analysed the relationship between this factor and microvessel count of the tumours. Although the mean microvessel count was higher in VEGF-positive compared with VEGF-negative tumours, no significant statistical difference was found. However, it is evident that the vascular phenotype in any one tumour is the result of a larger number of factors influencing angiogenesis; this process is driven by a complex array of soluble mediators, matrix molecules and accessory cells that function so as to fine tune and co-ordinate the response in both time and space. This prompts us to further analyse a spectrum of both stimulating and inhibiting angiogenic factors to better explain this process with particular attention to NSCLC.

Although many questions remain to be solved, the close association we found between VEGF mRNA expression and survival in NSCLC underlines the importance of the evaluation of this angiogenic factor in the biological assessment of this type of cancer. The analysis of this factor is potentially important to evaluate the angiogenic activity of tumours in order to develop new therapeutic strategies directed against biological targets.

### ACKNOWLEDGEMENT

This work has been supported by grants from the Associazione Italiana per la Ricerca sul Cancro.

### REFERENCES

Abu-Jawdeh GM, Faix JD, Niloff J, Tognazzi K, Manseau E, Dvorak HF and Brown LF (1996) Strong expression of vascular permeability factor (vascular endothelial growth factor) and its receptors in ovarian borderline and malignant neoplasms. *Lab Invest* **74**: 1105–1115

American Joint Committee on Cancer (1992) *Manual for a Staging of Cancer*. 4th edn, Beahrs OH, Henson DE, Hutter RU, Myers MH (eds), pp. 115–122. JP Lipincott: Philadelphia PA

Angeletti CA, Lucchi M, Fontanini G, Mussi A, Chella A, Ribechini A, Vignati S and Bevilacqua G (1996) Prognostic significance of tumoral angiogenesis in completely resected late stage lung carcinoma (stage IIIA–N2). Impact of adjuvant therapies in a subset of patients at high risk of recurrence. *Cancer* **78**: 409–415

Brown LF, Berse B, Jackman RW, Tognazzi K, Manseau EJ, Dvorak HF and Senger DR (1993) Increased expression of vascular permeability factor (vascular endothelial growth factor) and its receptors in kidney and bladder carcinomas. *Am J Pathol* **143**: 1255–1262

Brown LF, Berse B, Jackman RW, Tognazzi K, Guidi AJ, Dvorak HF, Senger DR, Connoly JL and Schnitt SJ (1995) Expression of vascular permeability factor (vascular endothelial growth factor) and its receptors in breast cancer. *Hum Pathol* **26**: 86–91

Ferrara N (1995) The role of vascular endothelial growth factor in pathological angiogenesis. *Breast Cancer Res Treatment* **36**: 127–137

Fontanini G, Bigini D, Vignati S, Basolo F, Mussi A, Lucchi M, Chinè S, Angeletti CA, Harris AL and Bevilacqua G (1995) Microvessel count predicts metastatic disease and survival in non-small cell lung cancer. *J Pathol* **177**: 57–63

Fontanini G, Vignati S, Boldrini L, Chinè S, Silvestri V, Lucchi M, Mussi A, Angeletti CA and Bevilacqua G (1997) Vascular endothelial growth factor (VEGF) is associated with neovascularization and influences progression of non-small cell lung carcinoma (NSCLC). *Clin Cancer Res* **3**: 861–865

Harpole DH, Richards WG, Herndon JE and Sugarbaker DJ (1996) Angiogenesis and molecular biologic substaging in patients with stage I non-small cell lung cancer. *Ann Thorac Surg* **61**: 1470–1476

Houck KA, Ferrara N, Winer J, Cachianes G, LI B and Leung DW (1991) The vascular endothelial growth factor family: identification of a fourth molecular species and characterization of alternative splicing of RNA. *Mol Endocrinol* **5**: 1806–1814

Inoue K, Ozeki Y, Suganuma T, Sugiura Y and Tanaka S (1997) Vascular endothelial growth factor expression in primary esophageal squamous cell carcinoma. Association with angiogenesis and tumor progression. *Cancer* **79**: 206–213

Macchiarini P, Fontanini G, Hardin JM, Squartini F and Angeletti CA (1992) Relation of neovascularisation to metastasis of non-small-cell lung cancer. *Lancet* **340**: 45–46

Maeda K, Chung Y, Ogawa Y, Takatsuka S, Kang SM, Ogawa M, Sawada T and Sow M (1996) Prognostic value of vascular endothelial growth factor expression in gastric carcinoma. *Cancer* **77**: 858–863

Martiny-Baron G and Marmé D (1995) VEGF- mediated tumour angiogenesis: a new target for cancer therapy. *Curr Opinion Biotechnol* **6**: 675–680

Mattern J, Koomagi R and Volm M (1995) Vascular endothelial growth factor expression and angiogenesis in non-small cell lung carcinomas. *Int J Oncol* **6**: 1059–1062

Mattern J, Koomagi R and Volm M (1996) Association of vascular endothelial growth factor expression with intratumoral microvessel density and tumour cell proliferation in human epidermoid lung carcinoma. *Br J Cancer* **73**: 931–934

Ohta Y, Endo Y, Tanaka M, Shimizu J, Oda M, Hayashi Y, Watanabe Y and Sasaki T (1996) Significance of vascular endothelial growth factor messenger RNA expression in primary lung cancer. *Clin Cancer Res* **2**: 1411–1416

Shibuya M (1995) Role of VEGF-FLT receptor system in normal and tumor angiogenesis. *Adv Cancer Res* **67**: 281–316

Suzuki K. Hayashi N, Miyamoto Y, Yamamoto M, Ohkawa K, Ito Y, Sasaki Y, Yamaguchi Y, Nakase H, Noda K, Enomoto N, Arai K, Yamada Y, Yoshihara H, Tujimura T, Kawano K, Yoshikawa K and Kamada T (1996) Expression of vascular permeability factor/vascular endothelial growth factor in human hepatocellular carcinoma. *Cancer Res* **56**: 3004–3009

Takahashi Y, Kitadai Y, Bucana CD, Cleary KR and Ellis LM (1995) Expression of vascular endothelial growth factor and its receptors, KDR, correlates with vascularity, metastasis, and proliferation of human colon cancer. *Cancer Res* **55**: 3964–3968

Takahashi Y, Cleary KR, Mai M, Kitadai Y, Bucana CD and Ellis LM (1996) Significance of vessel count and vascular endothelial growth factor and its receptors (KDR) in intestinal type gastric cancer. *Clin Cancer Res* **2**: 1679–1684

Tischer E, Mitchell R, Hartman T, Silva M, Gospodarowicz D, Fiddes JC and Abraham JA (1991) The human gene for vascular endothelial growth factor. *J Biol Chem* **266**: 11947–11954

Toi M, Kondo S, Suzuki H, Yamamoto Y, Inada K, Imazawa T, Taniguchi T and Tominaga T (1996) Quantitative analysis of vascular endothelial growth factor in primary breast cancer. *Cancer* **77**: 1101–1106

Volm M, Koomagi R and Mattern J (1996*a*) Vascular endothelial growth factor and basic fibroblast growth factor in primary lung carcinomas and the incidence of metastasis. *Int J Oncol* **9**: 711–714

Volm M, Koomagi R and Mattern J (1996*b*) Interrelationships between microvessel density, expression of VEGF and resistance to doxorubicin of non-small lung cell carcinoma. *Anticancer Res* **16**: 213–218

Wizigmann-Voos S, Breier G, Risau W and Plate KH (1995) Up-regulation of vascular endothelial growth factor and its receptors in von Hippel–Lindau disease-associated and sporadic hemangioblastomas. *Cancer Res* **55**: 1358–1364

World Health Organization (1982) The World Health Organization. Histological typing of lung tumours. *Am J Clin Pathol* **77**: 123–136

Yoshiji H, Gomez DE, Shibuya M and Thorgeirsson UP (1996) Expression of vascular endothelial growth factor, its receptors, and other angiogenic factors in human breast cancer. *Cancer Res* **56**: 2013–2016

© Cancer Research Campaign 1999
